# Protective effects and possible mechanisms of catalpol against diabetic nephropathy in animal models: a systematic review and meta-analysis

**DOI:** 10.3389/fphar.2023.1192694

**Published:** 2023-08-09

**Authors:** Zhongmei Fu, Xiaojuan Su, Qi Zhou, Haoyue Feng, Rui Ding, Hejiang Ye

**Affiliations:** ^1^ Chengdu University of Traditional Chinese Medicine, Chengdu, China; ^2^ Hospital of Chengdu University of Traditional Chinese Medicine, Chengdu, China

**Keywords:** catalpol, diabetic nephropathy, preclinical systematic review, efficacy, mechanism

## Abstract

**Aim of the Study:** *Rehmannia glutinosa* is a core Chinese herbal medicine for the treatment of diabetes and diabetic nephropathy (DN). It has been used for the treatment of diabetes for over 1,000 years. Catalpol is the main active compound in Rehmannia roots. Current evidence suggests that catalpol exhibits significant anti-diabetic bioactivity, and thus it has attracted increasing research attention for its potential use in treating DN. However, no studies have systematically evaluated these effects, and its mechanism of action remains unclear. This study aimed to evaluate the effects of catalpol on DN, as well as to summarize its possible mechanisms of action, in DN animal models.

**Materials and Methods:** We included all DN-related animal studies with catalpol intervention. These studies were retrieved by searching eight databases from their dates of inception to July 2022. In addition, we evaluated the methodological quality of the included studies using the Systematic Review Center for Laboratory animal Experimentation (SYRCLE) risk-of-bias tool. Furthermore, we calculated the weighted standard mean difference (SMD) with 95% confidence interval (CI) using the Review Manager 5.3 software and evaluated publication bias using the Stata (12.0) software. A total of 100 studies were retrieved, of which 12 that included 231 animals were finally included in this review.

**Results:** As compared to the control treatment, treatment with catalpol significantly improved renal function in DN animal models by restoring serum creatinine (Scr) (*p* = 0.0009) and blood urea nitrogen (BUN) (*p* < 0.00001) levels, reducing proteinuria (*p* < 0.00001) and fasting blood glucose (FBG) (*p* < 0.0001), improving kidney indices (*p* < 0.0001), and alleviating renal pathological changes in the animal models. In addition, it may elicit its effects by reducing inflammation and oxidative stress, improving podocyte apoptosis, regulating lipid metabolism, delaying renal fibrosis, and enhancing autophagy.

**Conclusion:** The preliminary findings of this preclinical systematic review suggest that catalpol elicits significant protective effects against hyperglycemia-induced kidney injury. However, more high-quality studies need to be carried out in the future to overcome the methodological shortcomings identified in this review.

## 1 Introduction

The high prevalence of diabetes and its complications is a major global health concern (Zheng et al., 2018). The International Diabetes Federation (IDF) estimated that the number of people living with diabetes will reach 7.832 million by 2045 (Sun et al., 2022). The high prevalence of diabetes has markedly increased the incidence of diabetic nephropathy (DN), which has become a common cause of death in patients with diabetes and a major contributor to the global burden of disease (Alicic et al., 2017; Braunwald, 2019). DN is the most serious microvascular complication of diabetes, which common pathogenic mechanisms include glucose metabolism disturbances, changes in renal hemodynamics, cytokine abnormalities such as pro-inflammatory factors, pro-fibrotic factors, oxidative stress-related factors, etc., (Alicic et al., 2017; Tanaka et al., 2018; Chen et al., 2022). And the pathological features mainly include glomerular basement membrane thickening, extracellular matrix deposition, glomerular hypertrophy, mesangial matrix expansion, podocyte loss, nodular glomerulosclerosis and tubulointerstitial fibrosis (Tervaert et al., 2010). If not treated in time, DN can progress to end-stage renal disease (ESRD). Presently, DN is the main reason for the implementation of renal replacement therapy (Fu et al., 2019). In addition, diabetes-induced kidney disease is strongly associated with cardiovascular mortality (Afkarian et al., 2013). Early diagnosis and treatment can not only delay disease progression and prevent DN-related complications, such as cardiovascular and retinal diseases, but also improve patients’ quality of life and reduce burden. Currently, the standard management strategy for diabetic kidney disease (DKD) mainly includes strict blood glucose and blood pressure control and management of the renin-angiotensin system (RAS) blockade; however, the efficacy and safety of RAS blockade therapy remain controversial (Suissa et al., 2006; Sarafidis and Bakris, 2009). It has been shown ineffective in inhibiting the progression of DN to ESRD, and serious side effects such as AKI and hyperkalemia have been observed (Onuigbo, 2011). Thus, novel strategies for DN prevention need to be urgently developed.

Traditional Chinese medicines (TCMs) are effective in treating diabetes and its complications. There are over 1,000 varieties of plants used in the treatment of diabetes worldwide (Marles and Farnsworth, 1995), such as resveratrol (Hu H. C. et al., 2022), artemisinin and its derivatives (Feng et al., 2022), berberine (Hu S. et al., 2022) and quercetin (Hu T. et al., 2022), have been shown strong protective effect against diabetic kidney damage. *Rehmannia glutinosa* Libosch. (Dihuang), one of the famous “Four Huai medicines”, was originally described in the Shen Nong Ben Cao Jing and is believed to have nourishing effects on the Yin and kidney (Zhang et al., 2008). Text mining of ancient and modern texts found that Rehmannia is a core component in both ancient and modern Chinese medicine formulations for the treatment of diabetes and DN (Zhao et al., 2016; Wu, 2021), and has been used for these purposes for over 1,000 years (Bai et al., 2019). Catalpol, an iridoid glycoside, is the main active compound in Rehmannia roots, and was first isolated by Kitagawa Hiroshi in 1971 (Kitagawa et al., 1971). Related pharmacokinetic studies (Lu et al., 2009; Li-nan et al., 2012)showed that catalpol could pass the blood-brain barrier and has a potential to be orally administrated, could be absorbed quickly, and exhibits a higher absolute bioavailability and a relatively longer half-life. It has been extensively studied in many disease states and has been shown to exhibit several biological effects, including neuroprotective (Yang et al., 2020), cardiovascular protective (He et al., 2021), anticancer (Wang and Zhan-Sheng, 2018), hepato-protective, anti-inflammatory, antioxidant, and anti-diabetic effects (Xu et al., 2020; 2015; Bhattamisra et al., 2019).

Catalpol has been considered a promising drug candidate for treating diabetes and its complications (Zhang et al., 2015; Bai et al., 2019). Recent studies performed in DN animal models have reported that catalpol can improve diabetes-induced kidney damage. However, using the same indicators, different studies have shown different efficacy levels for catalpol. Relevant evidence from preclinical studies on DN with catalpol intervention is scarce. This may increase uncertainty as to the efficacy of catalpol in ameliorating hyperglycemia-induced renal injury. Moreover, catalpol is currently not used in the clinical treatment of DN. Systematically reviewing all available data from animal experiments will enhance the reliability and robustness of the results and help translate novel therapeutic strategies from experimental results to clinical application. Therefore, this study aimed to evaluate the currently available evidence on the reno-protective effects of catalpol in DN animal models and to summarize the possible mechanisms underlying these effects.

## 2 Materials and methods

We conducted this report based on the Preferred Reporting Items for Systematic Reviews and Meta-Analyses checklist. This study was registered with PROSPERO (registration number: CRD42023434480).

### 2.1 Search strategy

Two authors independently searched for relevant animal studies performed using catalpol in DN animal models in the following eight electronic databases: PubMed, Embase, Web of Science, Scopus, China National Knowledge Internet (CNKI), VIP Information Chinese Periodical Service Platform (VIP), Wanfang Data Knowledge Service Platform (Wanfang), and China Biology Medicine Disc (CBM). Any disagreement were resolved through discussion with the third author during the systematic search process. All animal studies published from the date of inception of the databases to July 2022 were searched without language restrictions. The main search terms used were “Diabetic Nephropathies,” “Nephropathies, Diabetic,” “Nephropathy, Diabetic,” “Diabetic Nephropathy,” “Diabetic Kidney Disease,” “Diabetic Kidney Diseases,” “Kidney Disease, Diabetic,” “Kidney Diseases, Diabetic,” “DN” for participants, and “catalpol” for intervention. The specific retrieval strategies are listed in Supplementary Table S1.

### 2.2 Study selection

#### 2.2.1 Inclusion criteria

(1) Participants: DN animal models, irrespective of differences in species, sex, and modeling method;
(2) Intervention: catalpol, irrespective of the dosage, timing, and frequency of administration;
(3) Comparison: the same dose and mode of administration of non-functional substances (such as water and normal saline) or no treatment;
(4) Outcomes: the primary outcome was the restoration of serum creatinine (Scr) levels, and the secondary outcomes were blood urea nitrogen (BUN) levels, proteinuria, kidney index (KI), pathological changes in renal tissues, fasting blood glucose (FBG), body weight, and the mechanism of action.

#### 2.2.2 Exclusion criteria

(1) The study was not an animal study or was performed *in vitro*, in humans, or *in silico*;
(2) The article was a case report, review, or comment;
(3) Non-DN animal models;
(4) Other treatment drugs or catalpol in combination with other therapies;
(5) The absence of a separate control group;
(6) No relevant outcomes reported;
(7) Duplicated data or publications.

### 2.3 Data extraction

The NoteExpress software (version 3.6.0) was used to manage the retrieved studies. After deduplication, two authors independently performed literature screening based on the inclusion and exclusion criteria. First, included studies were initially screened by reading their titles and abstracts to exclude irrelevant articles; the full texts of the studies were then systematically reviewed to evaluate whether they were suitable for this meta-analysis. Subsequently, the following details were extracted from selected studies: (1) the name of the first author and publication year; (2) characteristics of the study animals (sample size, species, age, sex, and weight); (3) information on the animal models, including method of establishment of the models and criteria for the determination of successful modeling; (4) information on the treatment and control groups (administration route, dosage, and duration of treatment); and (5) outcome indicators. For republished studies, those with the most outcomes were chosen. When outcomes were presented at different time points, we extracted data from the last time point. In addition, if a study used drug dose gradients, we combined the multiple dose groups into a single dose group. Furthermore, we used the GetData Graph Digitizer software (version 2.26) to extract data for result indicators displayed graphically. Any unclear results were clarified through discussion with the third author during the data extraction process.

### 2.4 Risk of bias assessment

The quality of the included studies was independently assessed by two researchers using the SYstematic Review Center for Laboratory animal Experimentation (SYRCLE) risk-of-bias tool, and this assessment covered the following areas: (1) sequence generation; (2) baseline characteristics; (3) allocation concealment; (4) random housing; (5) blinding (for animal breeders and researchers); (6) random outcome assessment; (7) blinding (for outcome evaluators); (8) incomplete outcome data; (9) selective outcome reporting; and (10) other sources of bias. Each of these items was scored at 1 point. Any disagreements arising during the evaluation were resolved through discussion with the third author.

### 2.5 Data synthesis and analysis

We used the RevMan software (version 5.3) for data analysis. All data extracted from the included studies were continuous variables. Therefore, we described the effect size for catalpol intervention using the standard mean difference (SMD) and 95% confidence interval (95% CI). In addition, we used Cochran’s Q statistic and *I* ^2^ to assess heterogeneity. *I* ^2^ > 50% and *P*_Q test_ < 0.1 were considered to indicate significant heterogeneity. In case of high heterogeneity, we conducted a meta-regression analysis (Stata software version 12.0) using species, modeling methods, route of administration, and duration of treatment to evaluate the influence of the variables or research characteristics on heterogeneity and the estimated effect size. Since meta-regression analysis is applicable to indicators with more than 10 studies, for indicators with a smaller number of studies, subgroup analysis was performed. Egger’s test was used to detect publication bias, and sensitivity analyses were performed to assess the stability of the studies.

## 3 Results

### 3.1 Study selection

**FIGURE 1:**
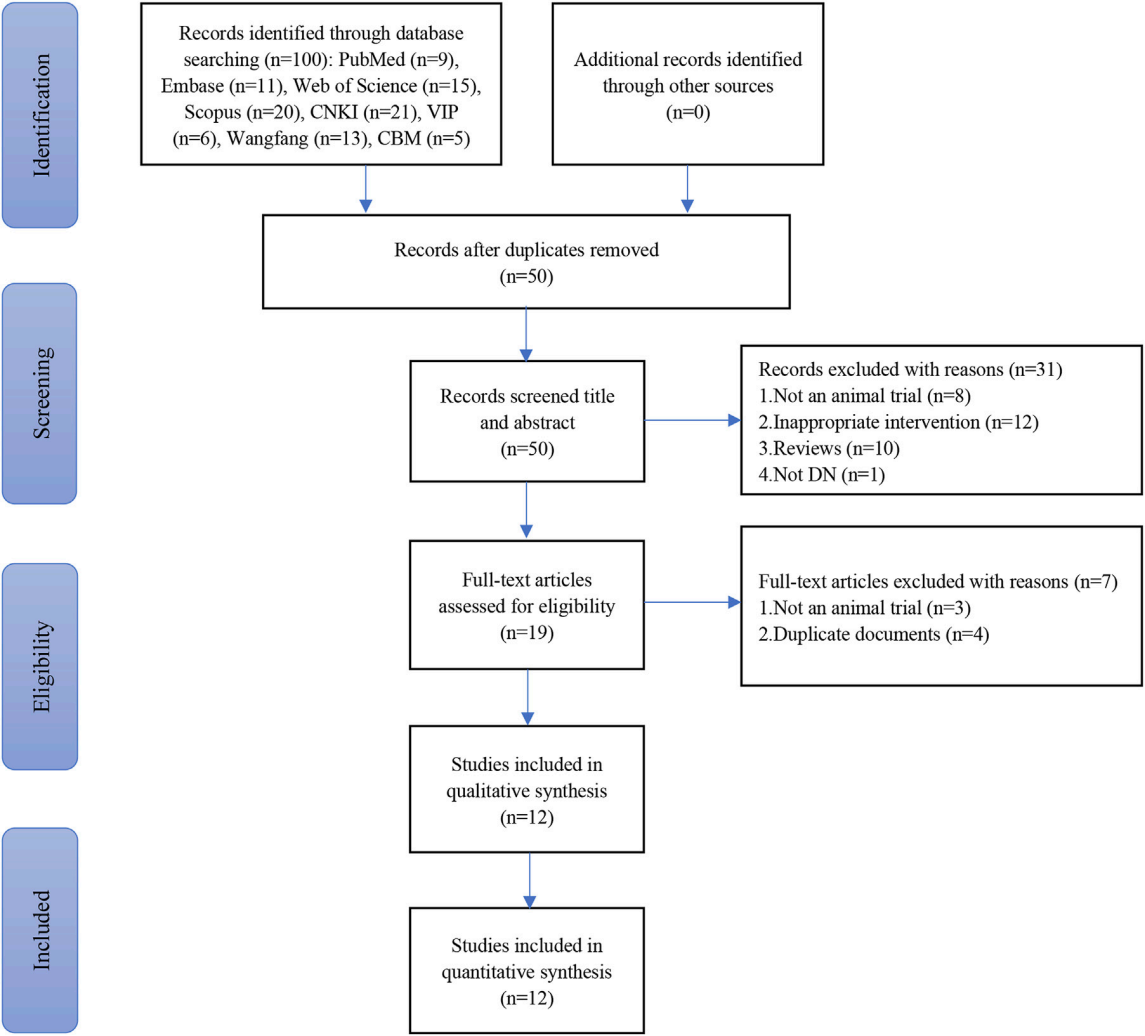
Flow diagram depicting the selection of studies.

One hundred articles were obtained from eight databases (8 from PubMed, 11 from Embase, 15 from Web of Science, 20 from Scopus, 21 from CNKI, 6 from VIP, 13 from Wanfang, and 5 from CBM). Of these, 50 were duplicates. Finally, 12 eligible articles were retrieved for this systematic review and meta-analysis (Zhou et al., 2012; Dong, 2013; Yang et al., 2016; Ding et al., 2017; Jiang et al., 2018; Chen X. F. et al., 2019; Chen Y. et al., 2019; Chen J. et al., 2020; Chen Y. et al., 2020; Meng et al., 2021; Shu et al., 2021; Zhang et al., 2022). The specific search process is shown in Figure 1.

### 3.2 Study characteristics

Twelve studies were included in this review. Of which, eight (Yang et al., 2016; Ding et al., 2017; Jiang et al., 2018; Chen X. F. et al., 2019; Chen Y. et al., 2019; Chen J. et al., 2020; Chen et al., 2020a; Shu et al., 2021) and four (Zhou et al., 2012; Dong, 2013; Meng et al., 2021; Zhang et al., 2022) were published in the English and Chinese languages, respectively. All 12 studies included male animals, with eight using mice (Yang et al., 2016; Jiang et al., 2018; Chen X. F. et al., 2019; Chen Y. et al., 2019; Chen J. et al., 2020; Chen et al., 2020a; Meng et al., 2021; Shu et al., 2021) and four using rats (Zhou et al., 2012; Dong, 2013; Ding et al., 2017; Zhang et al., 2022). Nine studies reported the animals’ ages (range: 4–12 weeks) (Yang et al., 2016; Ding et al., 2017; Jiang et al., 2018; Chen X. F. et al., 2019; Chen Y. et al., 2019; Chen J. et al., 2020; Chen Y. et al., 2020; Meng et al., 2021; Shu et al., 2021). Eight studies reported the animals’ body weights (Zhou et al., 2012; Dong, 2013; Yang et al., 2016; Ding et al., 2017; Jiang et al., 2018; Chen Y. et al., 2020; Meng et al., 2021; Zhang et al., 2022), with rats weighing 160–280 g and mice weighing 18–50 g. For the modeling methods, three studies adopted spontaneous diabetes models (Jiang et al., 2018; Chen J. et al., 2020; Shu et al., 2021). In six studies, DN was induced by intraperitoneal or tail vein streptozotocin (STZ) injection (Zhou et al., 2012; Yang et al., 2016; Ding et al., 2017; Chen X. F. et al., 2019; Meng et al., 2021; Zhang et al., 2022); in three studies, DN was induced by intraperitoneal STZ injection in combination with a high-fat diet (HFD) (Dong, 2013; Chen J. et al., 2020; Chen Y. et al., 2020). The dose of STZ ranged from 35 to 180 mg/kg. Blood glucose (BG) levels ≥16.7 mmol/L or FBG levels ≥11.1 mmol/L were adopted as criteria for the determination of successful modeling in seven studies (Dong, 2013; Yang et al., 2016; Ding et al., 2017; Chen X. F. et al., 2019; Chen J. et al., 2020; Meng et al., 2021; Zhang et al., 2022); however, this was not reported in five studies (Zhou et al., 2012; Yang et al., 2016; Chen Y. et al., 2019; Chen J. et al., 2020; Shu et al., 2021). As concerns catalpol administration methods, oral gavage was adopted in nine studies (Dong, 2013; Ding et al., 2017; Chen X. F. et al., 2019; Chen Y. et al., 2019; Chen J. et al., 2020; Chen et al., 2020a; Meng et al., 2021; Shu et al., 2021; Zhang et al., 2022) and intraperitoneal injection was adopted in two studies (Zhou et al., 2012; Yang et al., 2016). In one study, catalpol was mixed with food (Jiang et al., 2018). The drug dose ranged from 5 to 200 mg/kg/day, and dose gradients were adopted in five studies (Dong, 2013; Chen X. F. et al., 2019; Chen J. et al., 2020; Chen Y. et al., 2020; Shu et al., 2021). Scr and BUN levels were separately used as outcomes measures in 10 (Zhou et al., 2012; Dong, 2013; Yang et al., 2016; Ding et al., 2017; Jiang et al., 2018; Chen X. F. et al., 2019; Chen J. et al., 2020; Chen Y. et al., 2020; Shu et al., 2021; Zhang et al., 2022) and seven studies (Dong, 2013; Yang et al., 2016; Ding et al., 2017; Jiang et al., 2018; Chen J. et al., 2020; Chen Y. et al., 2020; Shu et al., 2021), respectively; proteinuria was separately reported as an outcome measures in eight studies (Dong, 2013; Yang et al., 2016; Ding et al., 2017; Jiang et al., 2018; Chen X. F. et al., 2019; Chen J. et al., 2020; Shu et al., 2021; Zhang et al., 2022), and urinary protein excretion was separately reported as an outcome measure in one study (Zhou et al., 2012). Furthermore, renal pathology was reported as an outcome measure in 11 studies (Zhou et al., 2012; Dong, 2013; Yang et al., 2016; Ding et al., 2017; Jiang et al., 2018; Chen X. F. et al., 2019; Chen J. et al., 2020; Chen Y. et al., 2020; Meng et al., 2021; Shu et al., 2021; Zhang et al., 2022), and KI were also reported in 11 studies (Zhou et al., 2012; Dong, 2013; Yang et al., 2016; Ding et al., 2017; Chen X. F. et al., 2019; Chen Y. et al., 2019; Chen J. et al., 2020; Chen et al., 2020a; Meng et al., 2021; Shu et al., 2021; Zhang et al., 2022); FBG levels were reported as an outcome measure in nine studies (Zhou et al., 2012; Yang et al., 2016; Ding et al., 2017; Jiang et al., 2018; Chen X. F. et al., 2019; Chen Y. et al., 2019; Chen J. et al., 2020; Chen Y. et al., 2020; Zhang et al., 2022). Body weight index was reported in six studies (Ding et al., 2017; Jiang et al., 2018; Chen X. F. et al., 2019; Chen J. et al., 2020; Chen Y. et al., 2020; Zhang et al., 2022). Some studies reported the changes in the levels of inflammatory markers, such as interleukin-6 (IL-6) (Chen Y. et al., 2020; Shu et al., 2021), interleukin-1β (IL-1β) (Ding et al., 2017; Chen J. et al., 2020; Meng et al., 2021; Shu et al., 2021), and tumor necrosis factor-(TNF-) α(Ding et al., 2017; Chen Y. et al., 2020; Shu et al., 2021). Some studies reported the changes in the levels of oxidative stress markers, such as superoxide dismutase (SOD) (Dong, 2013; Ding et al., 2017; Chen J. et al., 2020; Chen Y. et al., 2020) and malondialdehyde (MDA) (Dong, 2013; Chen J. et al., 2020). Furthermore, anti-apoptosis indicators such as caspase-3 (Yang et al., 2016; Ding et al., 2017), as well as representative anti-fibrosis indicators, such as TGF-β1, were also reported in some studies (Dong, 2013; Ding et al., 2017). The detailed characteristics of the included studies are shown in Table 1.

**TABLE 1:** Characteristics of the included studies.

Study	Species (sex, *n* = treatment/control group, age, weight)	Established model	Modeling standard	Experimental group (approach, daily dosage, duration)	Control group	Outcomes	Intergroup differences
Zhou et al. (2012)	SD rats (male, 6/6, NM, 200–250 g)	SIJ STZ (50 mg/kg)	NM	Intraperitoneal injection, 5 mg/kg/day, 2 weeks	Equal volume of NS	1.KI↓	1.*p > 0.05*
						2.Scr↓	*2.p > 0.05*
						3.FBG↓	*3.p > 0.05*
						4.UAER↓	*4.p < 0.05*
						5.IGF1 ↓	*5.p < 0.05*
						6.AKT ↓	6.*p < 0.05*
						7.P-AKT ↓	*7.p < 0.05*
						8.Renal pathology	
Dong (2013)	SD rats (male, 24/8, NM, 160–180 g)	HFD + SIJ STZ (35 mg/kg)	2 times RBG >16.7 mmol/L	Intragastric; 30, 60, and 120 mg/kg/day; 10 weeks	Equal volume of distilled water	1.KI↓	1.*p* < 0.01
						2.Scr↓	2.*p > 0.05*
						3.BUN↓	3.*p* < 0.01
						4.Proteinuria↓	4.*p* < 0.01
						5.RBG↓	5.*p* < 0.05
						6.GSP↓	6.*p* < 0.05
						7.Serum insulin↓	7.*p > 0.05*
						8.SOD↑	8.*p* < 0.05
						9.MDA↓	9.*p* < 0.01
						10.ANG-II↓ 11.TGF-β1↓	10.*p* < 0.01
						12.CTGF↓	11.*p* < 0.05
						13.FN↓	12.*p* < 0.01
						14.Col IV↓	13.*p* < 0.05
						15.Renal pathology	14.*p* < 0.01
Yang et al. (2016)	C57BL/6 mice (male, 7/6, 6–7 weeks, 20–22 g)	SIJ STZ (180 mg/kg)	BG > 16.7 mmol/L 72 h after STZ injection	Intraperitoneal injection, 10 mg/kg/day, 2 weeks	Equal volume of NS	1.KI↓	1.*p < 0.01*
						2.Scr↓	*2.p < 0.05*
						3.BUN↓4.Proteinuria	*3.p < 0.05*
						5.RBG↓	*4.p < 0.05*
						6.IGF-1↑	*5.p > 0.05*
						7.p-IGF-1R↑ 8.caspase3↓9.Grb10↓	6.*p < 0.05*
						10.Renal pathology	*7.p < 0.05*
							*8.p < 0.05*
							9.*p* < 0.05
Ding et al. (2017)	Wistar rats (male, 8/8, 12 weeks, 250 ± 30 g)	Single tail vein injection STZ (40 mg/kg)	BG > 16.7 mmol/L, urea sugar below “++” plus at least one-fold increase in urea volume 2 weeks after STZ injection	Intragastric, 20 mg/kg/day, 4 weeks	NM	1.KI↓	1.*p < 0.05*
						2.Scr↓	*2.p < 0.05*
						3.BUN↓	*3.p < 0.05*
						4.Proteinuria↓	*4.p < 0.05*
						5.FBG↓	*5.p < 0.05*
						6.Body weight↑	6.*p < 0.05*
						7.TNF-α↓	*7.p < 0.05*
						8.IL-1β↓	*8.p < 0.05*
						9.SOD↑10.LDH↓11.Caspase3↓	9.*p* < 0.05
						12.TGF-β1↓13.Renal pathology	10.*p < 0.05*
							11.*p* < 0.05
							12.*p < 0.05*
Chen et al. (2019a)	SPF C57BL/6 J mice (male, 8/8, 4 weeks, NM)	HFD + continuous STZ intraperitoneal injections for 3 days (70 mg/kg)	BG > 16.7 mmol/L	Intragastric, 200 mg/kg/day, 6 weeks	Equal volume of NS	1.KI↓	1.*p < 0.05*
						2.Scr↓	*2.p < 0.05*
						3.UREA↓	*3.p < 0.05*
						4.FBG↓	*4.p < 0.05*
						4.Serum albumin↓	*5.p < 0.05*
						6. Body weight↓	6.*p < 0.05*
						7.TG↓	*7.p < 0.01*
						8.TC↓	*8.p < 0.001*
						9.HDL-C↑10.LDL-C↑	9.*p* < 0.001
							10.*p > 0.05*
Jiang et al. (2018)	db/db mice (male, 6/6, 8 weeks, 40–50 g)	Spontaneous type 2 diabetes	NM	Regular diet supplemented with 100 mg/kg/day, 16 weeks	NM	1.Scr↓	1.*p* < 0.05
						2.BUN↓	2.*p* < 0.05
						3. Proteinuria↓	3.*p* < 0.05
						4.FBG↓	4.*p* < 0.05
						5.Body weight↑	5.*p* < 0.05
						6.blood uric acid↓	6.*p* > 0.05
						7.TG↓	7.*p* < 0.05
						8.TC↓	8.*p* > 0.05
						9.HDL-C↑ 10.LDL-C↓	9.*p* < 0.05
						11.Renal pathology	10.*p* > 0.05
Chen et al. (2019b)	C57BL/6J mice (male, 24/8, 8 weeks, NM)	SIJ STZ (170 mg/kg)	NM	Intragastric; 30, 60, and 120 mg/kg/day; 8 weeks	NM	1.KI↓	1.*p* < 0.05
						2.Creatinine clearance↑	2.*p* > 0.05
						3.Proteinuria↓	3.*p* < 0.05
						4.FBG↓	4. *p* > 0.05
						5.podocalyxin↓	5.*p* < 0.05
						6.synaptopodin↓	6.*p* < 0.05
						7.LC3B↑	7.*p* < 0.05
						8.P62↓	8.*p* < 0.05
						9.p-p70s6k↓10.TFEB↑11.Renal pathology	9.*p* < 0.05
							10.*p* < 0.05
Chen et al. (2020a)	KK-Ay mice (male, 12/6, 14 weeks, NM)	Spontaneous diabetes + HFD	NM	Intragastric; 50 and 100 mg/kg/day; 8 weeks	NM	1.KI↓	*1.p < 0.01*
						2.Scr↓	*2.p < 0.01*
						3.BUN↓	*3.p < 0.01*
						4.Proteinuria↓	*4.p < 0.05*
						5.FBG↓	*5.p < 0.01*
						6.GSP↓	*6.p < 0.05*
						7. Serum insulin↑	*7.p < 0.01*
						8.Body weight↑	*8.p < 0.01*
						9.SOD↑ 10.ROS↓	*9.p < 0.01*
						11.WT1↑	*10.p < 0.01*
						12.nephrin protein↑	*11.p < 0.05*
						13.BAX↓	*12.p < 0.05*
						14.Bcl-2↑	*13.p < 0.01*
						15.AGES↓	*14.p < 0.05*
						16.RAGE↓	*15.p < 0.01*
						17.p-p38MAPK↓	*16.p < 0.05*
						18.p-p65NF-κB↓	*17.p < 0.01*
						19.NOX4↓	*18.p < 0.01*
							*19. p < 0.01*
Chen et al. (2020b)	C57B/6 mice (male, 24/12, 8 weeks, 20 ± 2 g)	HFD + Continuous STZ intraperitoneal injections three times (60 mg/kg)	FBG >11.1 mmol/L	Intragastric; 100 and 200 mg/kg/day; 4 weeks	Equal volume of NS	1.KI	*1.p < 0.01*
						2.Scr	*2.p < 0.01*
						3.BUN	*3.p < 0.01*
						4.FBG	*4.p < 0.01*
						5.Body weight↑	*5.p < 0.01*
						6.IL-6↓	*6.p < 0.01*
						7.TNF-α↓	*7.p < 0.01*
						8.IL-1β↓	*8.p < 0.01*
						9.MDA↓	*9.p < 0.01*
						10.SOD↑	*10.p < 0.01*
						11.GSH-PX↑	*11.p < 0.01*
						12.P-AMPK↑	*12.p < 0.01*
						13.AMPK↑	*13.p < 0.01*
						14.SIRT1↑	*14.p < 0.01*
						15.P-NF-κB↓	*15.p < 0.05*
						16.ASC↓	*16.p < 0.01*
						17.NLRP3↓	*17.p < 0.01*
						18.GSDMD-N↓	*18.p < 0.01*
						19.Cleaved IL-1β↓	*19.p < 0.01*
						20.Cleaved caspase1↓	*20.p < 0.05*
						21.Renal pathology	
Shu et al. (2021)	KK-Ay mice (male, 12/6, 8 weeks, NM)	Spontaneous type 2 diabetes + HFD	NM	Intragastric; 50 and 100 mg/kg/day; 8 weeks	Equal volume of NS	1.KI↓	*1.p < 0.01*
						2.Scr↓	*2.p < 0.01*
						3.BUN↓	*3.p < 0.01*
						4.Proteinuria↓	*4.p < 0.01*
						4.VEGF↓	*5.p < 0.01*
						5.VE-Cadherin↓	*6.p < 0.01*
						6.ICAM-1↓	*7.p < 0.01*
						7.MCP-1↓	*8.p < 0.01*
						8.IL-6↓	*9.p < 0.01*
						9.TNF-α↓	*10.p < 0.01*
						10.IL-1β↓	*11.p < 0.01*
						11.RAGE↓	*12.p < 0.01*
						12.RhoA↓	*13.p < 0.01*
						13.ROCK↓	*14.p < 0.01*
						14.CD68↓	
						15.Renal pathology	
Meng et al. (2021)	C57BL/6 mice (male, 8/8, 7 weeks, 18–22 g)	STZ (100 mg/kg)	FBG >11.1 mmol/L at 3, 7, and 14 days, respectively, after STZ injection	Intragastric, 250 mg/kg/day, 4 weeks	Pure water	1.KI↑	*1.p > 0.05*
						2.IL-1β↓	*2.p > 0.05*
						3.TLR4↓	*3.p > 0.05*
						4.NF-KB↓	*4.p < 0.01*
						5.NLRP3↓	*5.p < 0.01*
						6.P-AMPK↑	*6.p < 0.01*
						7.Renal pathology	
Zhang et al. (2022)	SD rats (male, 8/8, NM, 180–210 g)	SIJ STZ (60 mg/kg)	FBG >16.7 mmol/L at 72 h after STZ injection	Intragastric, 10 mg/kg/day, 6 weeks	NS (2 mL/day)	1.KI↓	1.*p* < 0.01
						2.Scr↓	2.*p* < 0.01
						3.Proteinuria↓	3.*p* < 0.01
						4. FBG↓	4.*p* < 0.01
						5.Body weight↑	5.*p* < 0.01
						6.S100A8↓	6.*p* < 0.01
						7.TLR4↓	7.*p* < 0.01
						8.NF-κB↓	8.*p* < 0.01
						9.Renal pathology	

Abbreviations: AMPK, AMP-activated protein kinase; RBG, random blood glucose; FBG, fasting blood glucose; BUN, blood urea nitrogen; CTGF, connective tissue growth factor; GSH-Px, glutathione peroxidase; HFD, high-fat diet; KI: kidney index; LC3B, microtubule-associated proteins 1A/1B light chain 3B type II; p-MAPK, phospho-mitogen-activated protein kinase; MCP-1, monocyte chemoattractant protein-1; MDA, malondialdehyde; NF-*κ*B, nuclear factor kappa B; NM, not mentioned; NS, normal saline; p-Akt, phospho-protein kinase B; RhoA, ras-homolog gene family, member A; ROCK, rho-associated coiled-coil containing protein kinase; SCr, serum creatinine; SD rats, Sprague Dawley rats; SIJ, single intraperitoneal injection; SOD, superoxide dismutase; STZ, streptozotocin; TGF-*β*1, transforming growth factor-*β*1; TLR4, toll-like receptor 4; TNF-*α*, tumor necrosis factor-*α*; UAER, urinary albumin excretion rates; IGF-1, insulin-like growth factor 1; p-IGF-1R, insulin-like growth factor 1 receptor phosphorylation; GSP, glycated serum protein; ANG-II, angiotensin II; Col IV, collagen typeIV; FN, fibronecti; Grb10, growth factor receptor-bound protein 10; LDH, lactate hydrogenase; TC, total cholesterol; TG, triglyceride; HDL-C, high density lipoprotein cholesterol; LDL-C, low density lipoprotein cholesterol; AGE, advanced glycation end products; RAGE, receptor for advanced glycation end products; ROS, reactive oxygen species; NOX4, NADPH oxidase 4; NLRP3, NOD-like receptor pyrin domain-containing protein 3; Caspase1: cysteiny-1 aspartate specific protease; AMPK, adenosine 5′-monophosphate (AMP)-activated protein kinase; SIRT1, silencing information regulator related enzyme 1; ICAM-1, intercellular cell adhesion molecule-1; VE-Cadherin, vascular endothelial cadherin; VEGF, vascular endothelial growth factor; Bcl-2, B-cell lymphoma-2; Bax, bcl2-associated x. Compared with the control group, ↓ indicates reduction while ↑ indicates increase.

### 3.3 Risk of bias and quality of the included studies

Two authors assessed the risk of bias for the 12 included studies. Only one study described the method of random allocation generation (Dong, 2013), with 10 studies not mentioning the specific randomization methods (Zhou et al., 2012; Yang et al., 2016; Ding et al., 2017; Chen X. F. et al., 2019; Chen Y. et al., 2019; Chen J. et al., 2020; Chen et al., 2020a; Meng et al., 2021; Shu et al., 2021; Zhang et al., 2022) and one not applying randomization (Jiang et al., 2018). Similar baseline characteristics were reported between groups in three studies (Jiang et al., 2018; Chen J. et al., 2020; Shu et al., 2021). The use of allocation concealment among the different groups was not clearly described in all studies. Except for two studies that did not report on housing and environmental conditions (Zhou et al., 2012; Zhang et al., 2022), in all studies, animal housing conditions were the same. None of the studies reported on whether blinding was performed for animal breeders and investigators. As concerns the randomization of outcome assessments, all studies were classified as having a high risk. In addition, no study assessed the exact risk associated with the blinding of outcome measures. As pertains to incomplete outcome data, three studies were classified as having a high risk (Zhou et al., 2012; Dong, 2013; Yang et al., 2016). All included studies reported on all metrics described in the pre-specified study protocol, and no other sources of bias were noted. The complete quality assessment of the included studies is shown in Table 2

**TABLE 2:** Risk of bias of included studies.

Study	(1)	(2)	(3)	(4)	(5)	(6)	(7)	(8)	(9)	(10)	Scores
Zhou et al. (2012)	U	U	U	U	U	N	U	N	Y	Y	2
Dong (2013)	Y	U	U	Y	U	N	U	N	Y	Y	4
Yang et al. (2016)	U	U	U	Y	U	N	U	N	Y	Y	3
Ding et al. (2017)	U	U	U	Y	U	N	U	Y	Y	Y	4
Chen et al. (2019a)	U	U	U	Y	U	N	U	Y	Y	Y	4
Jiang et al. (2018)	N	Y	U	Y	U	N	U	Y	Y	Y	5
Chen et al. (2019b)	U	U	U	Y	U	N	U	Y	Y	Y	4
Chen et al. (2020a)	U	Y	U	Y	U	N	U	Y	Y	Y	5
Chen et al. (2020b)	U	U	U	Y	U	N	U	Y	Y	Y	4
Shu et al. (2021)	U	Y	U	Y	U	N	U	Y	Y	Y	5
Meng et al. (2021)	U	U	U	Y	U	N	U	Y	Y	Y	4
Zhang et al. (2022)	U	U	U	U	U	N	U	Y	Y	Y	3

(1) Sequence generation (2) baseline characteristics (3) allocation concealment (4) random housing (5) blinding (performance bias) (6) random outcome assessment (7) blinding (detection bias) (8) incomplete outcome data (9) selective outcome reporting (10) other sources of bias. Y: yes; N: no; U: unclear.

### 3.4 Effects of catalpol on kidney function

**FIGURE 2:**
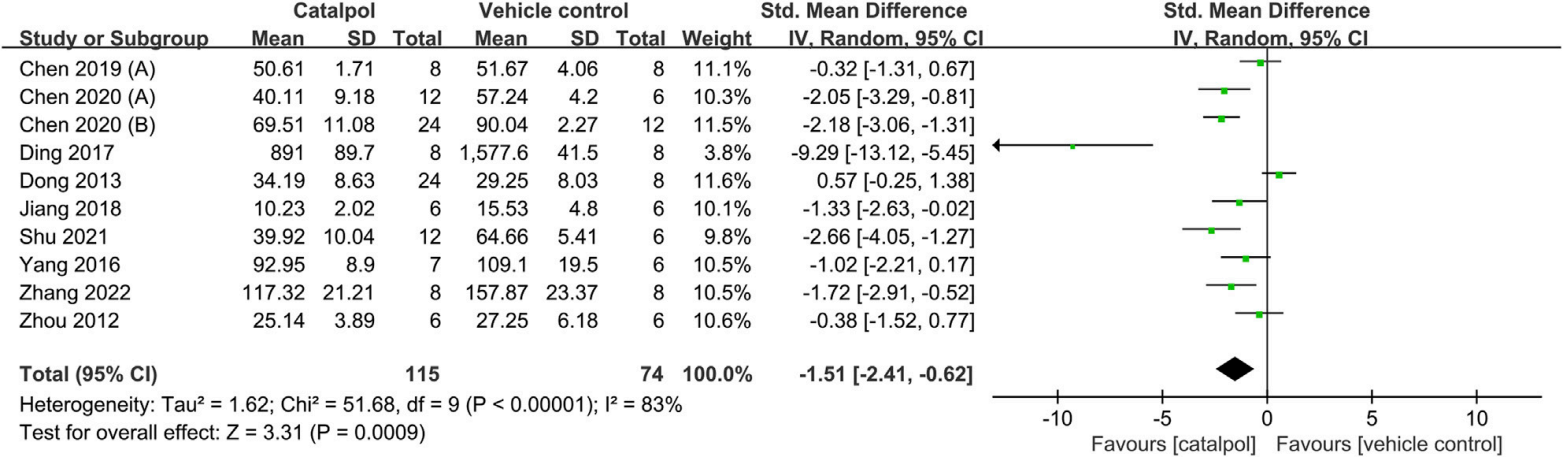
Forest plot showing the pooled effect estimation of catalpol on Scr.

Ten of the 12 studies reported Scr levels as outcome measures (Zhou et al., 2012; Dong, 2013; Yang et al., 2016; Ding et al., 2017; Jiang et al., 2018; Chen X. F. et al., 2019; Chen J. et al., 2020; Chen Y. et al., 2020; Shu et al., 2021; Zhang et al., 2022). The overall outcomes indicated that treatment with catalpol could effectively reduce Scr levels as compared to the vehicle or blank model group treatment SMD = −1.51, 95% CI: −2.41 to −0.62, *p* = 0.0009; heterogeneity: *I* ^2^ = 83%; *P*_Q test_ < 0.00001, Figure 2. We investigated potential variables that could increase heterogeneity through a meta-regression analysis. We found that species, modeling methods, route of administration, and duration of treatment were not sources of heterogeneity between the studies. The details of meta-regression analysis is shown in Table 3.

**TABLE 3:** The results of meta-regression analysis.

Parameter	Variable	Coefficient	t	*p*-Value	95% CI
Serum creatinine	Species	0.319	0.2	0.85	−3.45,4.09
	Modeling methods	0.231	0.24	0.816	−1.99,2.45
	Route of administration	1.27	0.68	0.513	−3.01,5.55
	Duration of treatment	0.599	0.38	0.713	−3.02,4.22
KI	Species	−1.952	−0.96	0.36	−6.53,2.63
	Modeling methods	−1.393	−1.14	0.285	−4.16,1.38
	Route of administration	3.036	1.27	0.236	−2.37,8.44
	Duration of treatment	0.557	0.27	0.794	−4.14,5.25

KI: kidney index.

**FIGURE 3:**
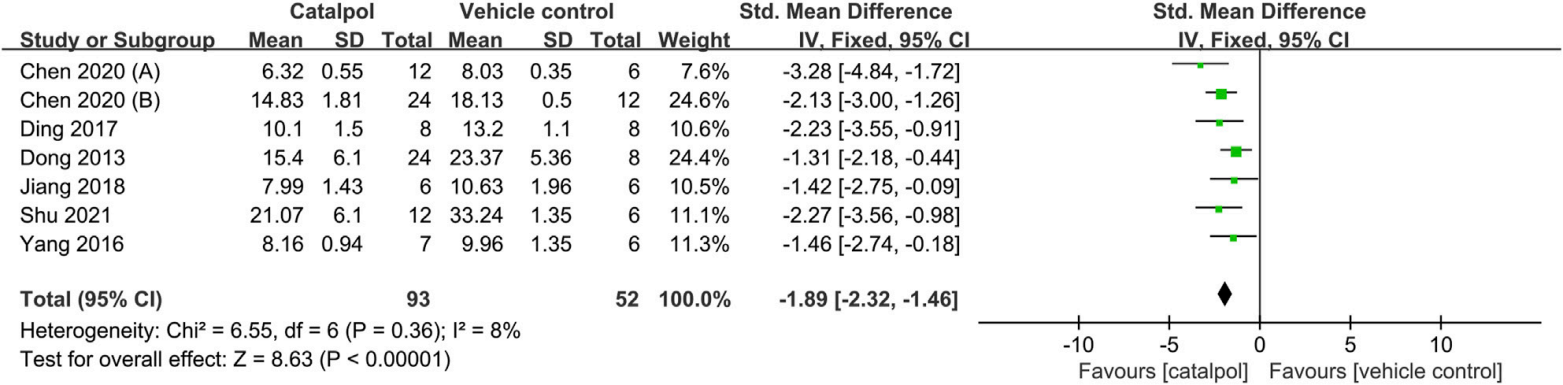
Forest plot showing the pooled effect estimation of catalpol on BUN.

Data from seven animal studies were pooled to assess the effects of catalpol on BUN levels (Dong, 2013; Yang et al., 2016; Ding et al., 2017; Jiang et al., 2018; Chen J. et al., 2020; Chen Y. et al., 2020; Shu et al., 2021). As heterogeneity was lower than 50% (*I* ^2^ = 8%, *P*_Q test_ = 0.36), a fixed-effects model was adopted. Our findings indicated that catalpol exerted significant beneficial effects in decreasing BUN levels as compared to the control treatment SMD = −1.89, 95% CI: −2.32 to −1.46, *p* < 0.00001, Figure 3.

**FIGURE 4:**
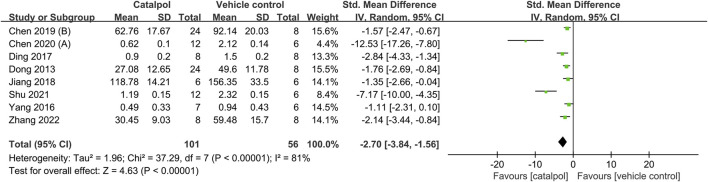
Forest plot showing the pooled effect estimation of catalpol on proteinuria.

The proteinuria-reducing effects of catalpol in DN animal models was assessed in eight animal studies (Dong, 2013; Yang et al., 2016; Ding et al., 2017; Jiang et al., 2018; Chen X. F. et al., 2019; Chen J. et al., 2020; Shu et al., 2021; Zhang et al., 2022). It was found to induce a significant decrease in proteinuria SMD = −2.70, 95% CI: −3.84 to −1.56, *p* < 0.00001; heterogeneity: *I* ^2^ = 81%, *P*_Q test_ < 0.00001, Figure 4. The results of the subgroup analysis for proteinuria showed no significant difference with respect to species (SMD: −2.08 VS. −3.62; *p* > 0.05), modeling method (SMD: −1.79 VS. -6.68 VS. −1.76; *p* > 0.05), and duration of treatment (SMD: −1.91 VS. −3.17; *p* > 0.05). However, subgroup differences resulting from the administration route could not be determined due to the limited number of studies. The details of subgroup analysis and forest plot is shown in Table 4; Supplementary Figure S1. In addition, urinary albumin excretion rates were only reported in one study (Zhou et al., 2012), in which catalpol was found to reduce these rates.

**TABLE 4:** The results of meta-regression analysis.

Comparison	Subgroup	No. of studies	SMD [95% CI]	*P* For meta-analysis	*I* ^2^	*P* For heterogeneity
Proteinuria						
Modeling methods	STZ	4	−1.79 [−2.44, −1.14]	<0.00001	17%	0.3
	Spontaneous diabetes	3	−6.68 [−12.77, −0.60]	0.03	93%	<0.00001
	STZ + HFD	1	−1.76 [−2.69, −0.84]	0.002	—	—
Route of administration	Intragastric	7	−3.04 [−4.36, −1.73]	<0.00001	83%	<0.00001
	Intraperitoneal injection	1	−1.11 [−2.31, 0.10]	0.07	—	—
Duration of treatment	≤6 weeks	2	−1.91 [−3.60, −0.22]	0.03	68%	0.08
	>6 weeks	6	−3.17 [−4.70, −1.64]	<0.0001	85%	<0.00001
Species	Rat	3	−2.08 [−2.75, −1.41]	<0.00001	0	0.49
	Mice	5	−3.62 [−5.74, −1.50]	0.0008	89	<0.00001
FBG						
Modeling methods	STZ	5	−1.21 [−2.16, −0.27]	0.01	70	0.01
	Spontaneous diabetes	2	−3.32 [−4.56, −2.09]	<0.00001	0	0.47
	STZ + HFD	2	−10.59 [−17.47, −3.71]	0.003	80	0.03
Route of administration	Intragastric	7	−3.31 [−5.13, −1.48]	0.0004	90	<0.00001
	Intraperitoneal injection	2	−0.42 [−1.21, 0.38]	0.31	0	0.70
Duration of treatment	≤6 weeks	5	−3.03 [−5.99, −0.07]	0.04	94	<0.00001
	>6 weeks	4	−2.04 [−3.32, −0.76]	0.002	75	0.008
Species	Rat	3	−1.79 [−3.65, 0.08]	0.06	83	0.003
	Mice	6	−2.99 [−5.14, −0.85]	0.006	92	<0.00001

### 3.5 Effects of catalpol on renal pathology

**FIGURE 5:**
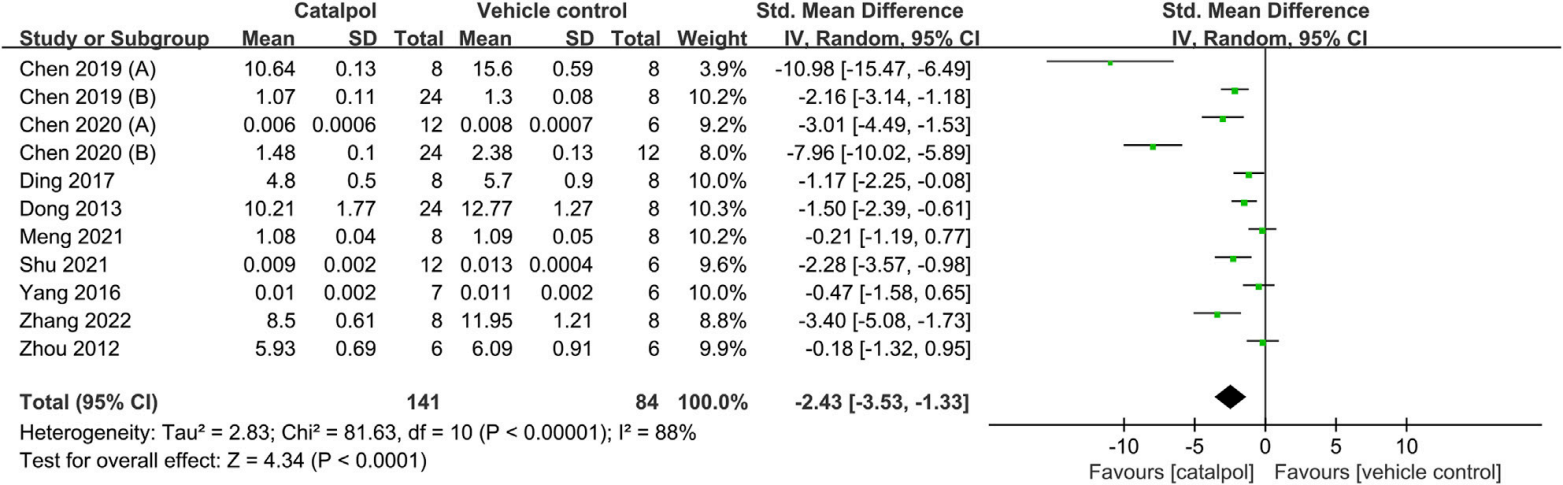
Forest plot showing the pooled effect estimation of catalpol on KI.

Data on the effects of catalpol in comparison to the control treatment on the kidney index was reported in 11 animal studies (Zhou et al., 2012; Dong, 2013; Yang et al., 2016; Ding et al., 2017; Chen X. F. et al., 2019; Chen Y. et al., 2019; Chen J. et al., 2020; Chen Y. et al., 2020; Meng et al., 2021; Shu et al., 2021; Zhang et al., 2022). As compared to the control treatment, treatment with catalpol significantly decreased the kidney index SMD = −2.43, 95% CI: −3.53 to 1.33, *p* < 0.0001; heterogeneity: *I* ^2^ = 88%; *P*_Q test_ < 0.00001, Figure 5. The meta-regression analysis showed that species, modeling method, route of administration, and duration of treatment were not sources of heterogeneity between the studies. The details of meta-regression analysis is shown in Table 3.

Six studies (Zhou et al., 2012; Dong, 2013; Jiang et al., 2018; Chen X. F. et al., 2019; Chen J. et al., 2020; Meng et al., 2021) reported that catalpol alleviated membrane cell proliferation and membrane matrix broadening. In addition, catalpol was reported to inhibit the thickening of the substrate membrane in six studies (Zhou et al., 2012; Dong, 2013; Chen J. et al., 2020; Meng et al., 2021; Shu et al., 2021; Zhang et al., 2022). Three studies (Chen J. et al., 2020; Meng et al., 2021; Zhang et al., 2022) reported that catalpol could improve glomerular expansion, and three other studies reported that it improved glomerulosclerosis (Zhou et al., 2012; Chen J. et al., 2020; Meng et al., 2021). Catalpol was reported to significantly ameliorate foot process effacement in three studies (Jiang et al., 2018; Chen X. F. et al., 2019; Chen Y. et al., 2020); in another study (Chen J. et al., 2020; Chen Y. et al., 2020; Shu et al., 2021), it was found to significantly improve vacuolar degeneration in renal tubular epithelial cells. In addition, it was reported to alleviate glomerular fibrosis in four studies (Yang et al., 2016; Jiang et al., 2018; Chen J. et al., 2020; Shu et al., 2021). Moreover, four of the animal studies (Jiang et al., 2018; Chen J. et al., 2020; Chen Y. et al., 2020; Shu et al., 2021) reported that catalpol alleviated renal damage by lowering glomerular regional glycogen deposition. Periodic acid-Schiff (PAS) staining, which was performed in one study (Dong, 2013), showed that catalpol decreased the integrated optical density of the glomerulus.

### 3.6 Effects of catalpol on FBG levels and weight

**FIGURE 6:**
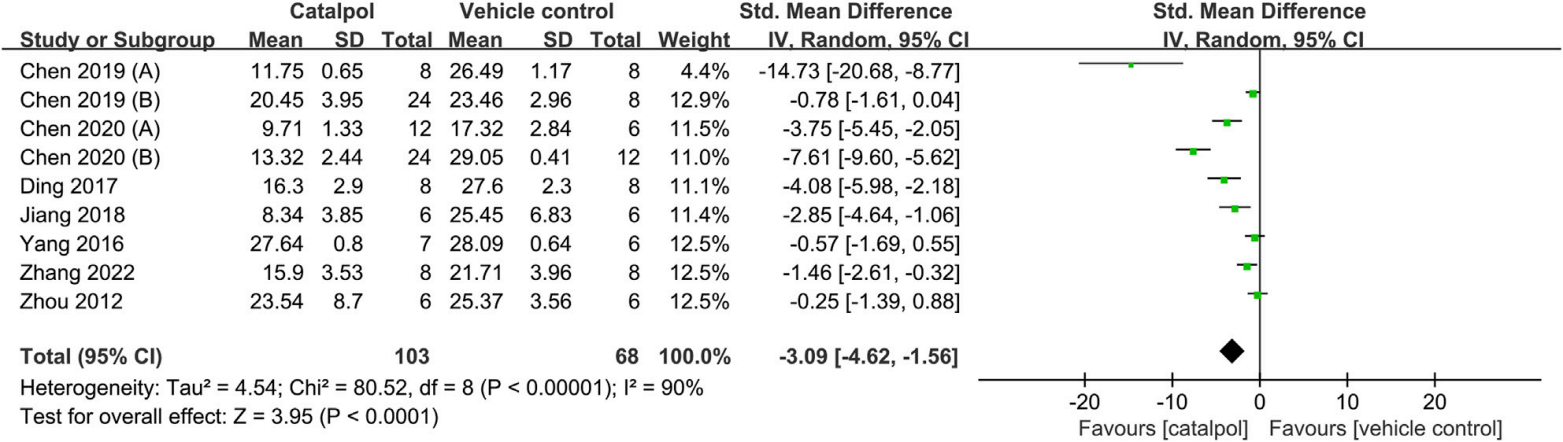
Forest plot showing the pooled effect estimation of catalpol on FBG.

Nine studies provided quantitative data on FBG levels (Zhou et al., 2012; Yang et al., 2016; Ding et al., 2017; Jiang et al., 2018; Chen X. F. et al., 2019; Chen et al., 2019a; Chen J. et al., 2020; Chen Y. et al., 2020; Zhang et al., 2022). The pooled results indicated that as compared to the control treatment, catalpol significantly decreased FBG levels (*n* = 171; SMD = −3.09, 95% CI: −4.62 to −1.56], *p* < 0.0001; heterogeneity: *I* ^2^ = 90%; *P*_Q test_ < 0.00001, Figure 6). The subgroup analysis showed no significant differences in the effects of catalpol with respect to animal species (SMD: −1.79 VS. −2.99; *p* > 0.05) and duration of treatment (SMD: −3.03 VS. −2.04; *p* > 0.05). As concerns modeling methods, the administration of STZ in combination with HFD showed better effects than the spontaneous diabetes method and intraperitoneal STZ injection alone (SMD: −10.59 VS. −3.32 VS. −1.21; *p* < 0.05). The heterogeneity of the studies was reduced. In addition, intragastric administration was associated with better effects than intraperitoneal injection (SMD: −3.42 VS. −0.42; *p* < 0.05), and heterogeneity was slightly reduced. The details of subgroup analysis and forest plots is shown in Table 4; Supplementary Figure S1. Further, two studies (Dong, 2013; Chen J. et al., 2020) reported that catalpol lowered glycated serum protein (GSP) levels.

**FIGURE 7:**
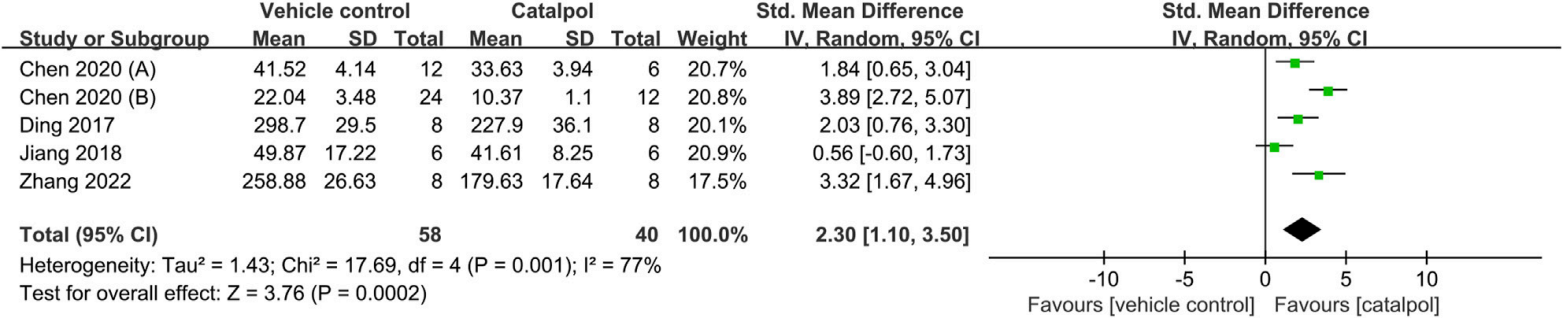
Forest plot showing the pooled effect estimation of catalpol on body weight.

Body weight was reported in six studies (Ding et al., 2017; Jiang et al., 2018; Chen X. F. et al., 2019; Chen J. et al., 2020; Chen Y. et al., 2020; Zhang et al., 2022), one of which was excluded due to the unavailability of data (Chen Y. et al., 2019). An overall analysis of the five studies retained showed that body weight was higher in DN animals treated with catalpol than in animals that received the control treatment (*n* = 98; SMD = 2.3, 95% CI: 1.1 to 3.5; *p* = 0.0002; heterogeneity: *I* ^2^ = 77%; *P*_Q test_ = 0.001, Figure 7).

### 3.7 Mechanism indicators

**FIGURE 8:**
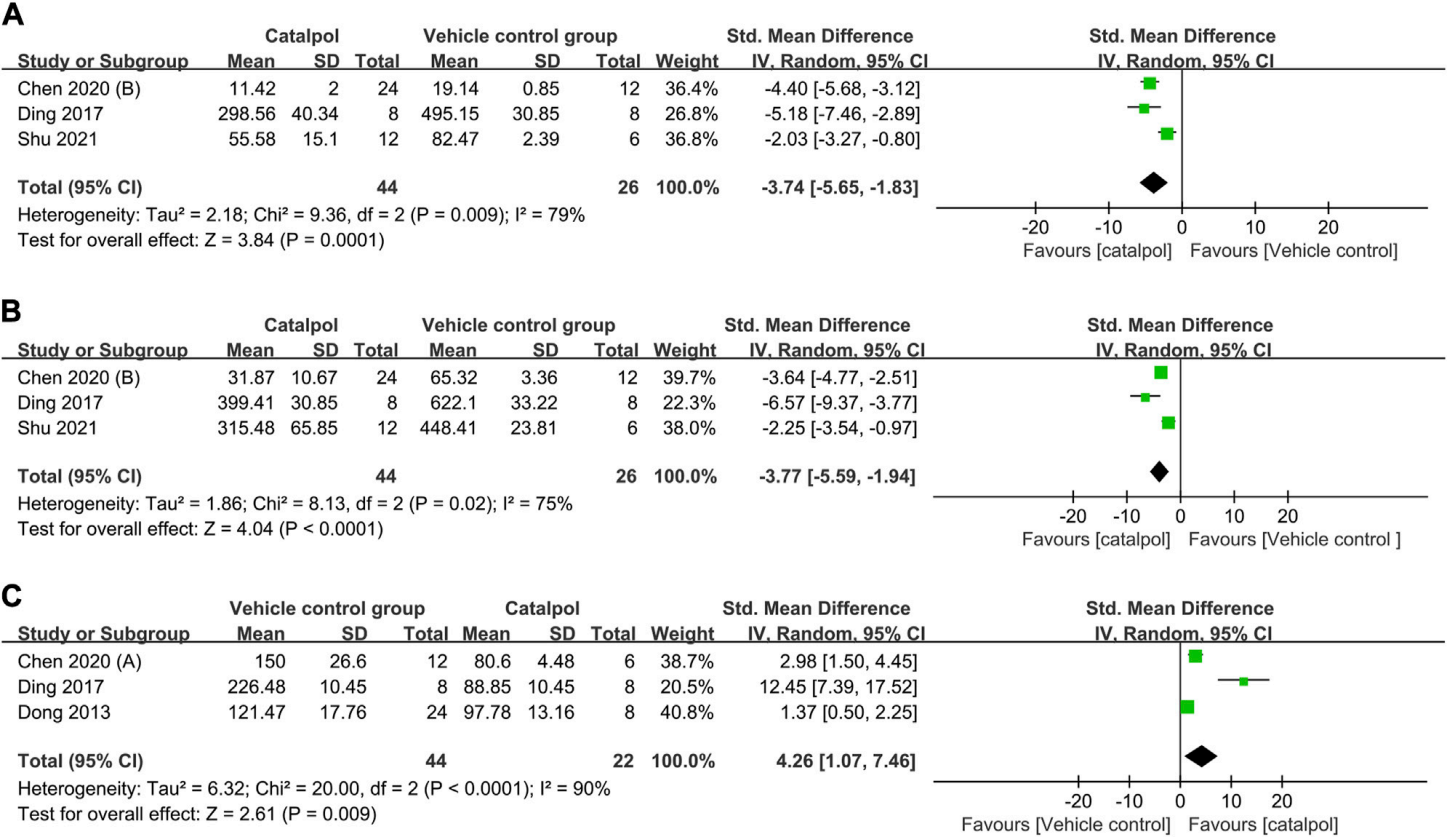
Forest plot showing the pooled effect estimation of catalpol on **(A)** serum IL-1β level, **(B)** serum TNF-α levels, and **(C)** renal SOD levels.

(1) Anti-inflammatory mechanism: Three studies (Ding et al., 2017; Chen J. et al., 2020; Shu et al., 2021) reported on serum interleukin (IL)-1β levels, and their findings indicated that treatment with catalpol significantly decreased IL-1β levels (*n* = 70; SMD = −3.74, 95% CI: −5.65 to −1.83); *P* = 0.0001; heterogeneity: *I* ^2^ = 79%; *P*_Q test_ = 0.009, Figure 8A). Two studies (Chen Y. et al., 2020; Meng et al., 2021) reported that catalpol decreased renal IL-1β levels (*P* < 0.01). A meta-analysis of the findings of three studies (Ding et al., 2017; Chen J. et al., 2020; Shu et al., 2021) showed a significant decrease in TNF-α levels following treatment with catalpol (n = 70; SMD = −3.77, 95% CI: −5.59 to −1.94]; *P* < 0.0001; heterogeneity: *I* ^2^ = 75%; *P*_Q test_ = 0.02, Figure 8B). Two studies (Chen J. et al., 2020; Shu et al., 2021) reported that catalpol decreased serum IL-6 levels (*P* < 0.01).
(2) Antioxidant mechanism: A meta-analysis of the results of 3 studies (Dong, 2013; Ding et al., 2017; Chen Y. et al., 2020) showed that compared to the control treatment, treatment with catalpol was associated with higher renal tissue (*n* = 66; SMD = 4.26, 95% CI: 1.07 to 7.46]; *P* = 0.009; heterogeneity: *I* ^2^ = 90%; *P*_Q test_ < 0.0001, Figure 8C) and serum (*P <* 0.01) SOD protein levels. One study (Chen J. et al., 2020) reported that catalpol raised serum SOD levels (*P* < 0.01).

### 3.8 Sensitivity analysis and publication bias

The sensitivity analysis of the primary outcome indicator, Scr, suggested no significant difference in the pooled effect size following the exclusion of each study from the meta-analysis. The lowest and highest combined effects were −1.19 (95% CI: −1.94–−0.43) and −1.72 (95% CI: −2.55–−0.9), respectively, after excluding the studies by Ding et al. and Dong et al.

**FIGURE 9:**
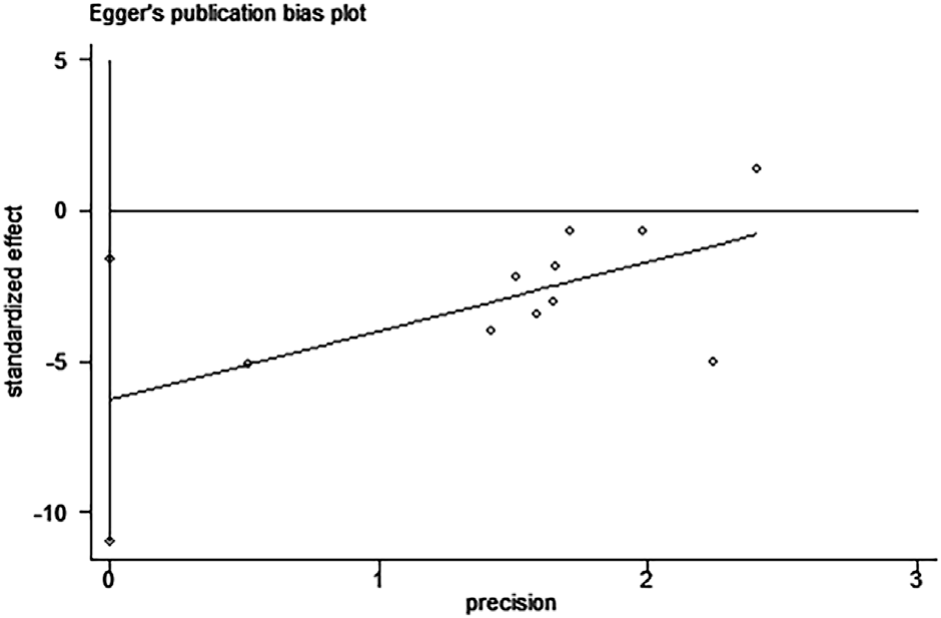
Egger’s publication bias plot for Scr.

**FIGURE 10:**
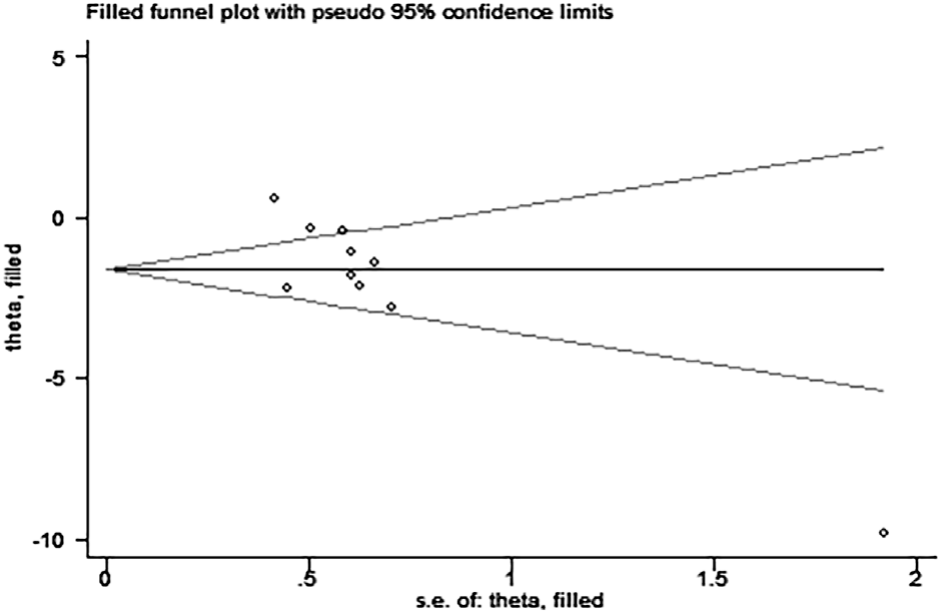
Trim and fill analysis for Scr.

Egger’s test revealed the possibility of publication bias for Scr (*P >* ItI = 0.015, Figure 9). Based on the random effects model, there was no supplemental dummy study and no change in the pooled effect of catalpol on Scr through the trim-and-fill analysis (Figure 10).

## 4 Discussion

### 4.1 Efficacy of catalpol

To the best of our knowledge, ours is the first preclinical systematic review to evaluate the renoprotective effects of catalpol in DN animal models, as well as its possible mechanism of action. An analysis of 12 studies that included 231 animals showed that catalpol significantly improved renal function indices, such as Scr, BUN, and urine protein levels, lowered blood sugar levels, and alleviated renal pathological changes. In addition, its potential mechanisms of action include the promotion of anti-inflammatory and antioxidant processes, alleviation of podocyte apoptosis, regulation of lipid metabolism, delay of fibrosis, and promotion of autophagy. Notably, treatment with catalpol significantly decreased FBG levels but increased body weight compared to control animals, which should be considered with regard to the conflicting results in metabolic diseases.

### 4.2 Implications

A decline in renal function is one of the main diagnostic criteria for DN. Scr and BUN levels are the most important indicators that reflect the state of renal function. Our meta-analysis showed that catalpol significantly improves renal function in DN animal models. Since our meta-regression analysis showed that the species, modeling method, treatment method, and treatment time were not sources of heterogeneity, we did not conduct further subgroup analysis for Scr. Although Egger’s test revealed the existence of possible publication bias, the stability of our results was not affected by publication bias through the cut-and-fill method. Therefore, we speculated that the heterogeneity observed probably resulted from complex pathological features of the disease and other differences between the studies, including differences in the inconsistent baseline level, STZ injection dose and frequency, and the standard for the determination of successful modeling. Thus, we recommend that the design, implementation, and reporting of the findings of future preclinical studies should strictly follow the relevant guidelines, such as the Animal Research: Reporting of *In Vivo* Experiments (ARRIVE) (Kilkenny et al., 2012) or Harmonized Animal Research Reporting Principles (HARRP) (Osborne et al., 2018) guidelines, to improve the reliability of animal research and its applicability to humans. In addition, dose-response and time-response effects play an important role in determining the effectiveness of clinical treatments. By studying the available literature, only a few of the included studies were found to use catalpol dose gradients (Dong, 2013; Chen X. F. et al., 2019; Chen J. et al., 2020; Chen et al., 2020a; Shu et al., 2021); four studies assessed the effects of different catalpol doses on Scr levels (Dong, 2013; Chen J. et al., 2020; Chen Y. et al., 2020; Shu et al., 2021). Three of these studies (Chen J. et al., 2020; Chen Y. et al., 2020; Shu et al., 2021)showed that catalpol decreased Scr levels in a dose-dependent manner, and one study (Dong, 2013) reported no significant differences in Scr levels between the different dose groups (*p* > 0.05). As concerns the duration of treatment, subgroup analysis showed no difference in Scr levels between the short (≤6 weeks) and long (>6 weeks) treatment periods. Presently, no study has reported on the optimal therapeutic dose and duration of treatment for catalpol. Therefore, determining the optimal dose and duration of treatment for catalpol is an important research direction for future studies.

Proteinuria is considered one of the most significant risk factors for loss of renal function (Sever and Schiffer, 2018), and restoring the level of proteinuria is considered an important therapeutic goal. The results of our meta-analysis suggest that catalpol treatment is significantly associated with lower levels of protein in urine. Existing evidence suggests that catalpol potentially alleviates proteinuria by improving podocyte injury. Podocytes are now considered to play a critical role in the development of several forms of proteinuric nephropathy, including DN (Assady et al., 2019). Chen et al. (Chen J. et al., 2020)suggested that catalpol could improve podocyte dysfunction by restoring levels of the podocyte injury molecules WT1 and nephrin, which are lytic membrane proteins in podocytes; they also reported that it could maintain the integrity of the filtration membrane and ensure proper podocyte function. Furthermore, the podocyte injury-improving effects of catalpol are mainly related to its anti-apoptosis and autophagy-inducing effects (Chen X. F. et al., 2019; Chen J. et al., 2020). In summary, catalpol can alleviate proteinuria and protect renal function through several mechanisms.

Proper glycemic control is a fundamental strategy for preventing DN development and progression. As early as 1971, Kitagawa Hiroshi demonstrated the hypoglycemic effects of catalpol (Kitagawa et al., 1971). In recent years, increasing evidence corroborating the notion that catalpol has potential anti-diabetic effects has been published. Yan et al. (Yan et al., 2018)found that catalpol improves insulin sensitivity and alleviates insulin resistance by decreasing IRS-1 phosphorylation and increasing AKT phosphorylation through the activation of the AMPK pathway. The results of this meta-analysis also indicated that catalpol could significantly reduce FBG levels in DN animal models. Functional impairment of pancreatic β cells and insulin resistance are the main risk factors of type 2 diabetes. The studies we included all used the type 2 diabetes model. Therefore, we speculate that this may be related to the effect of catalpol on improving insulin resistance. In addition, subgroup analysis showed that, as compared to the control treatment, treatment with catalpol could effectively reduce FBG levels following three modeling methods; however, the injection of STZ in combination with HFD showed better effects than the injection of STZ alone or the spontaneous diabetes method. In addition, heterogeneity was decreased among studies, suggesting that the hypoglycemic effects of catalpol may be affected by different modeling methods. However, there is not enough evidence to support the conclusion that STZ injection in combination with HFD exerts better effects in reducing FBG levels than STZ injection and the spontaneous diabetes method. The catalpol dose associated with STZ injection in combination with HFD was generally higher than that associated with the other two methods, which may have affected the extent to which FBG levels decreased. Xue et al. have reported the highest concentration and distribution of catalpol in the kidney following oral administration (Xue et al., 2015), which provides a material basis for DN treatment using catalpol. The subgroup analysis carried out in this study also showed that catalpol was more effective in reducing FBG levels following intragastric administration than the control treatment, indicating that catalpol can be administered orally.

The analysis of body weight yielded interesting results. As is well known, obesity is closely related to the development of DN, and weight loss can significantly improve glucose homeostasis and kidney injury by reducing lipotoxicity (Opazo-Ríos et al., 2020). In this study, the body weights of animals treated with catalpol were found to be significantly higher than those of animals subjected to the control treatment. In addition, similar results have been obtained in other animal disease models, such as the cuprizone (CPZ)-induced demyelination model (Sun et al., 2021) and the chronic unpredictable mild stress (CUMS)-induced depression model (Wang et al., 2021). A diabetic rabbit model, established using a hyperlipidemic diet in combination with alloxan, also exhibited weight loss (Liu et al., 2016). However, catalpol administration did not significantly increase body weight in normal animals (Wang et al., 2021). Furthermore, in an HFD-induced liver steatosis obese mouse model, weight loss was observed following catalpol treatment. Therefore, it is unclear whether it was the improvement of the disease or catalpol that was responsible for the weight gain. The effects of catalpol on body mass index should be investigated in future human trials. If catalpol is still found to increase body weight in patients with diabetes or diabetic complications, its applicability should be further assessed.

The mechanisms by which catalpol exerts renoprotective effects are summarized as follows:

(1) Inhibition of kidney inflammation: Inflammatory factors, such as IL-1β, TNF-α, and IL-6, play a key role in the occurrence of DN-related kidney injury. Catalpol has been reported to alleviate kidney inflammation by inhibiting NF-κB activation and ASC expression, inhibiting NLRP3 inflammasome expression through the inhibition of the AMPK/SIRT1 pathway, and by directly decreasing the expression levels of inflammatory factors such as IL-1β and TNF-α(Chen J. et al., 2020; Shu et al., 2021).
(2) Inhibition of oxidative stress: Under hyperglycemic conditions, increased NADPH oxidase (Nox) and ROS expression cause renal damage (Caramori and Mauer, 2003). Existing evidence suggests that catalpol can alleviate renal oxidative damage by inhibiting NOX4 and MDA activity, decreasing ROS expression, and increasing GSH and SOD activity.
(3) Reduction of apoptosis: Chen et al. (Chen J. et al., 2020) found that catalpol could increase the expression levels of the anti-apoptotic protein Bcl-2 and decrease the expression levels of the pro-apoptotic proteins Bax and Caspase 3, thereby exerting protective effects on the kidney.
(4) Regulation of lipid metabolism: Dyslipidemia is a risk factor for DN development and progression. Catalpol can regulate total cholesterol (TC), triglyceride (TG), and high-density lipoprotein cholesterol (HDL-C) levels, thereby improving lipid homeostasis and reducing the burden on the kidneys; however, it does not affect low-density lipoprotein cholesterol (LDL-C) levels (Jiang et al., 2018; Chen X. F. et al., 2019).
(5) Delay of renal fibrosis: Catalpol has been reported to delay renal fibrosis by decreasing the expression levels of FN, col IV, and CTGF through the downregulation of ANG II and TGF-β1 protein and mRNA levels, and by increasing occludin and VE-cadherin protein expression to some extent (Dong, 2013; Ding et al., 2017; Shu et al., 2021). Moreover, using Masson and PAS staining, catalpol was found to decrease collagen fiber content, the degree of fibrosis, and glycogen deposition (Dong, 2013; Yang et al., 2016; Jiang et al., 2018; Chen J. et al., 2020; Chen Y. et al., 2020; Shu et al., 2021).
(6) Enhancement of autophagy: Catalpol was found to improve foot cell damage by enhancing autophagy, which was associated with the inactivation of mTORC1 and activation of the TFEB protein, as well with an increase in the expression levels of the autophagy marker, LC3B, and a decrease in the expression levels of p62 (Chen X. F. et al., 2019).

### 4.3 Limitations

This study had some limitations. First, the lack of core criteria for the study design, including randomization methods, allocation concealment, and baseline characteristics, may have led to an overestimation of the effects of catalpol in the included studies. In addition, it is important for quality control to be performed in the studies as pertains to the calculation of sample size and the blinding of outcome measures; however, these were not mentioned in the included studies. Second, only 12 studies were included in this review, and the sample size for some indicators was insufficient. Third, although we conducted a subgroup analysis, there was still some unexplained heterogeneity. Therefore, we recommend that further high-quality studies with larger sample sizes be carried out in the future to confirm our findings. Fourth, animals suffering from hypertension, heart disease, or other diabetes-related comorbidities that have a significant impact on disease prognosis and drug efficacy were not evaluated in the included studies.

## 5 Conclusion

Despite the low quality of studies included in this meta-analysis, as well as their small number, the results of our study suggest that catalpol can reduce blood glucose levels and improve renal function. In addition, its mechanisms of action are closely related to the induction of anti-inflammatory and antioxidation processes, delay in renal fibrosis, and regulation of lipid homeostasis. Catalpol has been applied in clinical practice for the treatment of cancer and showed benefits in clinical outcome, at low cost, and with no side effect. Furthermore, catalpol exists high bioavailability and good tolerability. To sum up, catalpol may be a promising drug candidate for investigation in clinical trials for the provision of novel management options for DN, should be further evaluated. A more robust experimental design needs to be applied in future studies.

## Figures and Tables

**Table:** 

**CKD**	chonic kidney disease
**ESRD**	end-stage renal disease
**DN**	diabetic nephropathy
**DKD**	diabetic kidney disease
**RAS**	renin-angiotensin system
**SMD**	standard mean difference
**AMPK**	AMP-activated protein kinase
**RBG**	random blood glucose
**FBG**	fasting blood glucose
**BUN**	blood urea nitrogen
**CTGF**	connective tissue growth factor
**GSH-Px**	glutathione peroxidase
**HFD**	high-fat diet; KI: kidney index
**LC3B**	microtubule-associated proteins 1A/1B light chain 3B type II
**p-MAPK**	phospho-mitogen-activated protein kinase
**MCP-1**	monocyte chemoattractant protein-1
**MDA**	malondialdehyde
**NF-*κ*B**	nuclear factor kappa B
**NM**	not mentioned
**NS**	normal saline
**p-Akt**	phospho-protein kinase B
**RhoA**	ras-homolog gene family, member A
**ROCK**	rho-associated coiled-coil containing protein kinase
**SCr**	serum creatinine
**SD rats**	Sprague Dawley rats
**SIJ**	single intraperitoneal injection
**SOD**	superoxide dismutase
**STZ**	streptozotocin
**TGF-*β*1**	transforming growth factor-*β*1
**TLR4**	toll-like receptor 4
**TNF-*α***	tumor necrosis factor-*α*
**UAER**	urinary albumin excretion rates
**IGF-1**	insulin-like growth factor 1
**p-IGF-1R**	insulin-like growth factor 1 receptor phosphorylation
**GSP**	glycated serum protein
**ANG-II**	angiotensin II
**Col IV**	collagen typeIV
**FN**	fibronecti
**Grb10**	growth factor receptor-bound protein 10
**LDH**	lactate hydrogenase
**TC**	total cholesterol
**TG**	triglyceride
**HDL-C**	high density lipoprotein cholesterol
**LDL-C**	low density lipoprotein cholesterol
**AGE**	advanced glycation end products
**RAGE**	receptor for advanced glycation end products
**ROS**	reactive oxygen species
**NOX4**	NADPH oxidase 4
**NLRP3**	NOD-like receptor pyrin domain-containing protein 3
**Caspase1**	cysteiny-1 aspartate specific protease
**AMPK**	adenosine 5′-monophosphate (AMP)-activated protein kinase
**SIRT1**	silencing information regulator related enzyme 1
**ICAM-1**	intercellular cell adhesion molecule-1
**VE-Cadherin**	vascular endothelial cadherin
**VEGF**	vascular endothelial growth factor
**Bcl-2**	B-cell lymphoma-2
**Bax**	bcl2-associated x
