# Supplementary material for: Protective effects and possible mechanisms of catalpol against diabetic nephropathy in animal models: a systematic review and meta-analysis
Source: Front Pharmacol. 2023 Aug 9;14:1192694. doi: 10.3389/fphar.2023.1192694 (PMC10446169; doi:10.3389/fphar.2023.1192694)
Supplement: Supplementary file 1 [file Table1.docx]

Search strategy for PubMed

| Number | Search terms |
| --- | --- |
| 1 | (("Diabetic Nephropathies"[Mesh]) OR ((((((((((((((((((Diabetic Nephropathies[Title/Abstract]) OR (Nephropathies, Diabetic[Title/Abstract])) OR (Nephropathy, Diabetic[Title/Abstract])) OR (Diabetic Nephropathy[Title/Abstract])) OR (Diabetic Kidney Disease[Title/Abstract])) OR (Diabetic Kidney Diseases[Title/Abstract])) OR (Kidney Disease, Diabetic[Title/Abstract])) OR (Kidney Diseases, Diabetic[Title/Abstract])) OR (Diabetic Glomerulosclerosis[Title/Abstract])) OR (Glomerulosclerosis, Diabetic[Title/Abstract])) OR (Intracapillary Glomerulosclerosis[Title/Abstract])) OR (Nodular Glomerulosclerosis[Title/Abstract])) OR (Glomerulosclerosis, Nodular[Title/Abstract])) OR (Kimmelstiel-Wilson Syndrome[Title/Abstract])) OR (Kimmelstiel Wilson Syndrome[Title/Abstract])) OR (Syndrome, Kimmelstiel-Wilson[Title/Abstract])) OR (Kimmelstiel-Wilson Disease[Title/Abstract])) OR (Kimmelstiel Wilson Disease[Title/Abstract]))) |
| 2 | catalpol[Title/Abstract] |
| 3 | 1 AND 2 |

Search strategy for Embase

| Number | Search terms |
| --- | --- |
| 1 | 'diabetic nephropathies':ab,ti OR 'nephropathies, diabetic':ab,ti OR 'nephropathy, diabetic':ab,ti OR 'diabetic nephropathy':ab,ti OR 'diabetic kidney disease':ab,ti OR 'diabetic kidney diseases':ab,ti OR 'kidney disease, diabetic':ab,ti OR 'kidney diseases, diabetic':ab,ti OR 'diabetic glomerulosclerosis':ab,ti OR 'glomerulosclerosis, diabetic':ab,ti OR 'intracapillary glomerulosclerosis':ab,ti OR 'nodular glomerulosclerosis':ab,ti OR 'glomerulosclerosis, nodular':ab,ti OR 'kimmelstiel-wilson syndrome':ab,ti OR 'kimmelstiel wilson syndrome':ab,ti OR 'syndrome, kimmelstiel-wilson':ab,ti OR 'kimmelstiel-wilson disease':ab,ti OR 'kimmelstiel wilson disease':ab,ti |
| 2 | catalpol:ab,ti |
| 3 | 1 AND 2 |

Search strategy for Web of science

| Number | Search terms |
| --- | --- |
| 1 | (((((((((((((((((TS=(Diabetic Nephropathies)) OR TS=(Nephropathies, Diabetic )) OR TS=(Nephropathy, Diabetic)) OR TS=(Diabetic Nephropathy )) OR TS=(Diabetic Kidney Disease )) OR TS=(Diabetic Kidney Diseases )) OR TS=(Kidney Disease, Diabetic)) OR TS=(Kidney Diseases, Diabetic )) OR TS=(Diabetic Glomerulosclerosis )) OR TS=(Glomerulosclerosis, Diabetic)) OR TS=(Intracapillary Glomerulosclerosis)) OR TS=(Nodular Glomerulosclerosis)) OR TS=(Glomerulosclerosis, Nodular )) OR TS=(Kimmelstiel-Wilson Syndrome )) OR TS=(Kimmelstiel Wilson Syndrome)) OR TS=(Syndrome, Kimmelstiel-Wilson)) OR TS=(Kimmelstiel-Wilson Disease)) OR TS=(Kimmelstiel Wilson Disease ) |
| 2 | TS=(catalpol) |
| 3 | 1 AND 2 |
