# Supplementary material for: Protective effects and possible mechanisms of catalpol against diabetic nephropathy in animal models: a systematic review and meta-analysis
Source: Front Pharmacol. 2023 Aug 9;14:1192694. doi: 10.3389/fphar.2023.1192694 (PMC10446169; doi:10.3389/fphar.2023.1192694)
Supplement: Supplementary file 2 [file DataSheet1.docx]

**(A)**


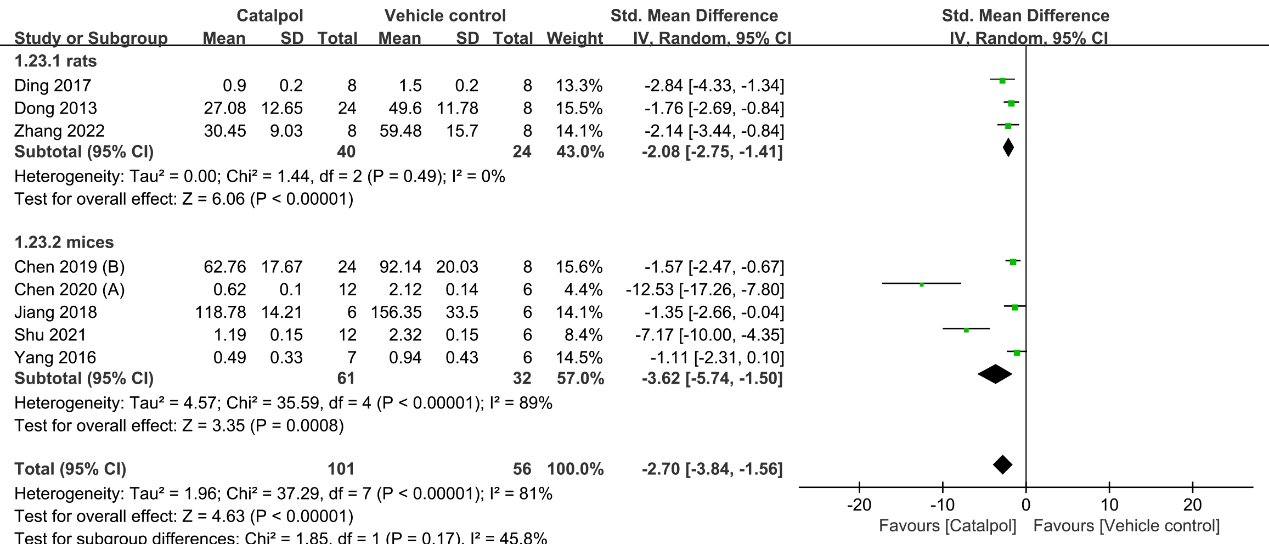


**(B)**


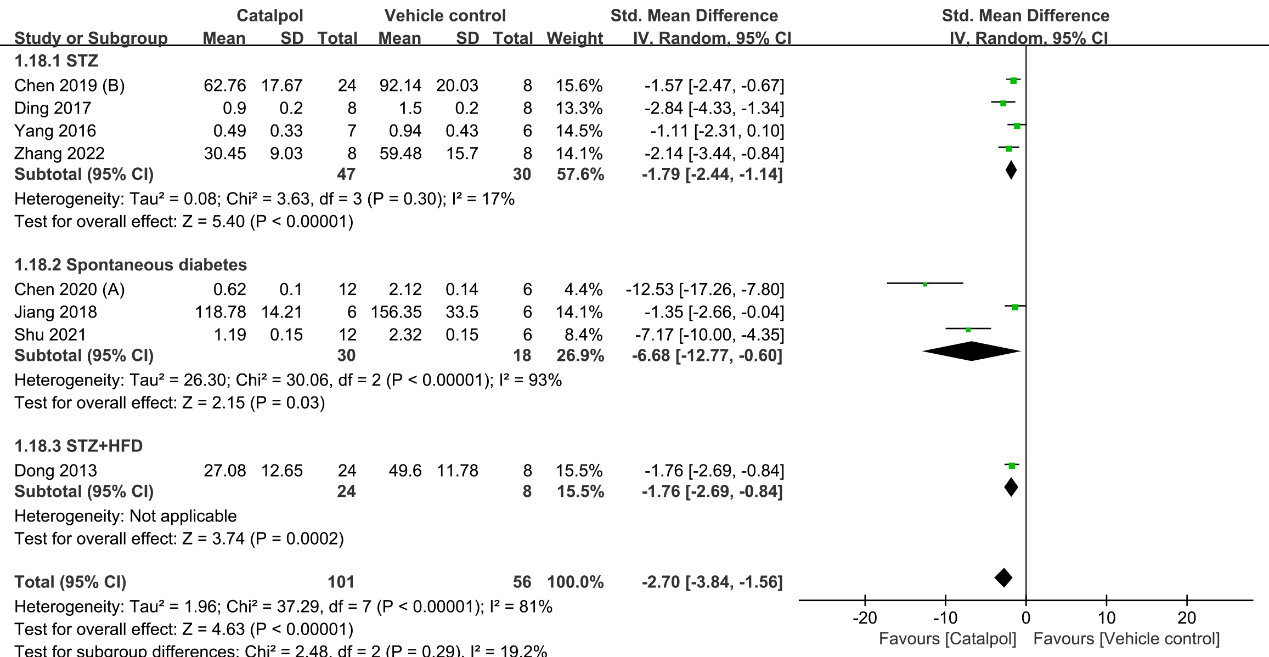


**(C)**


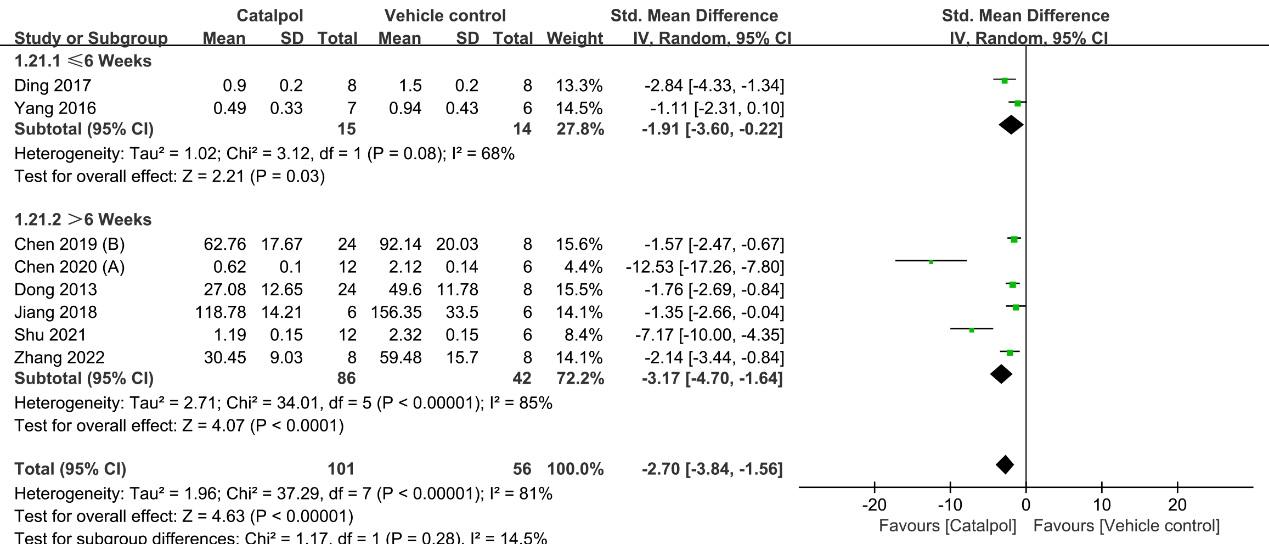


**Figure S1.** Effects of catalpol on proteinuria in animals with diabetic kidney disease (DKD) compared with vehicle control by the subgroup of different (A) species, (B) modeling methods, (C) duration of treatment. Abbreviations: CI, confidence interval; HFD, high-fat diet; IV, inverse variance; SD, standard deviation; STZ: streptozotocin.

**(A)**


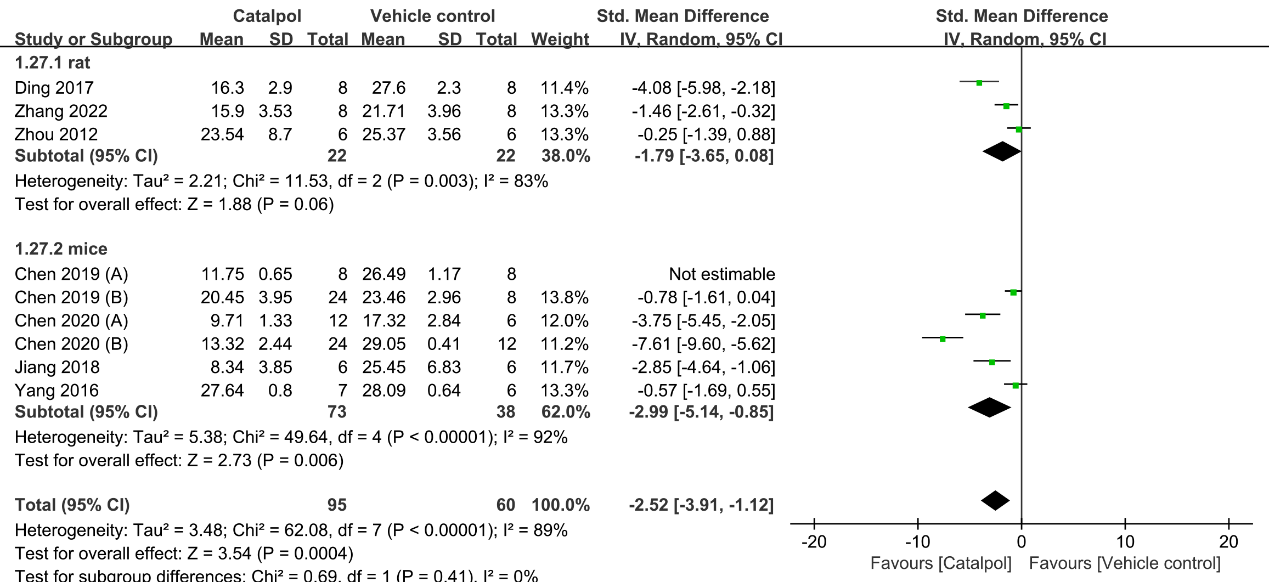


**(B)**

**
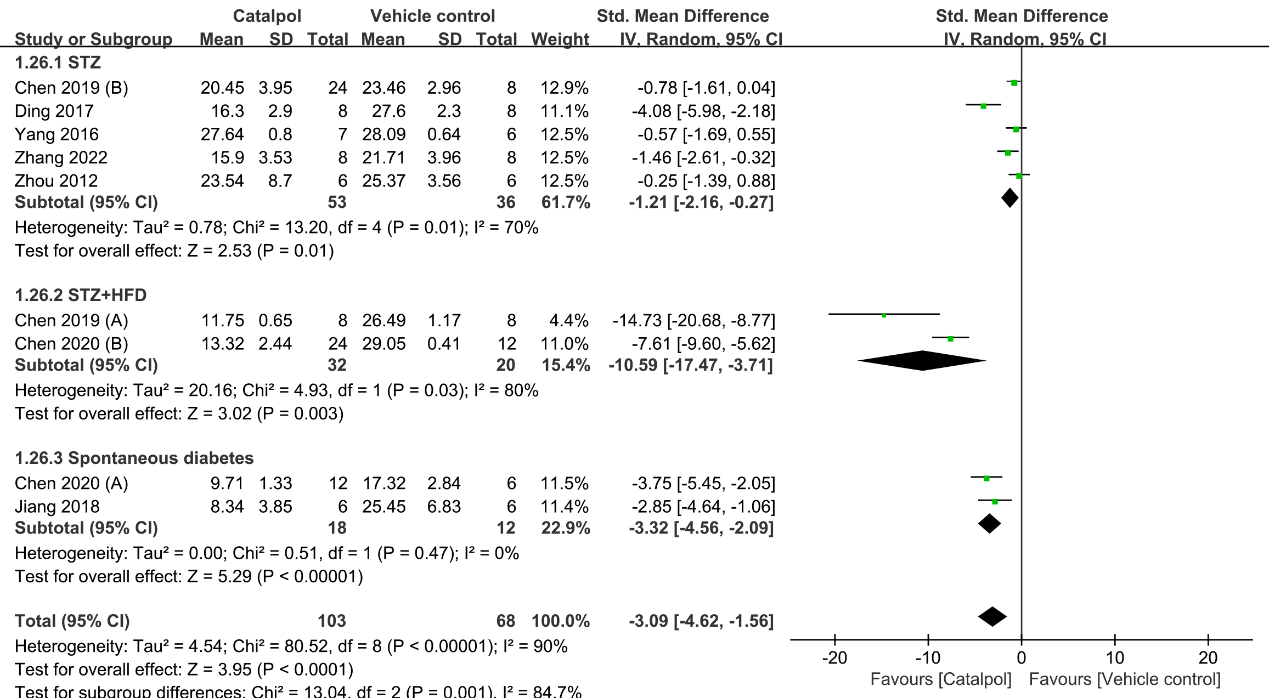
**

**(C)**


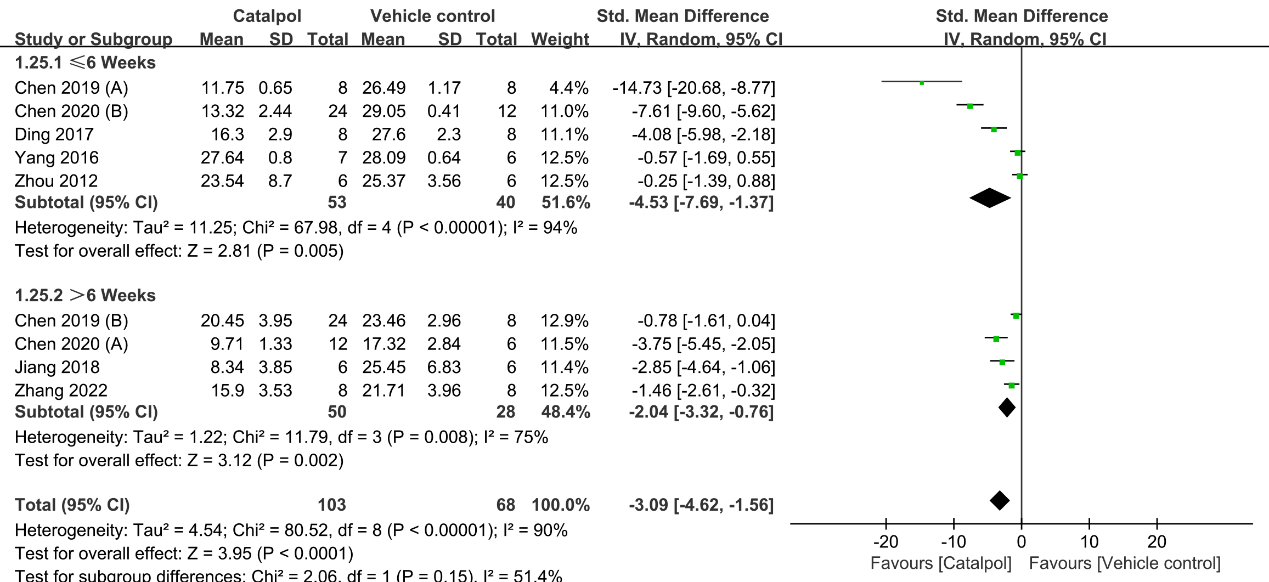


**(D)**


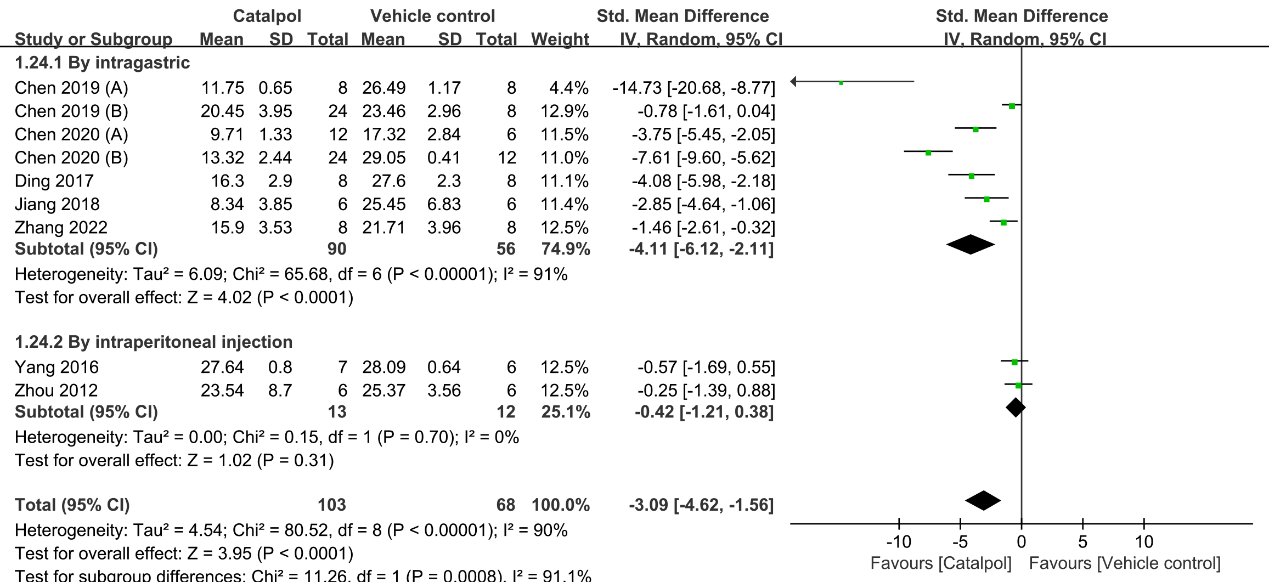


**Figure S1.** Effects of catalpol on FBG in animals with DN compared with vehicle control by the subgroup of different (A) species, (B) modeling methods and (C) duration of treatment, (D) route of administration. Abbreviations: FBG: fasting blood glucose; CI, confidence interval; HFD, high-fat diet; IV, inverse variance; SD, standard deviation; STZ: streptozotocin.
